# pwrEWAS: a user-friendly tool for comprehensive power estimation for epigenome wide association studies (EWAS)

**DOI:** 10.1186/s12859-019-2804-7

**Published:** 2019-04-29

**Authors:** Stefan Graw, Rosalyn Henn, Jeffrey A. Thompson, Devin C. Koestler

**Affiliations:** 1 Department of Biostatistics & Data Science, University of Kansas Medical Center, Kansas City, KS USA; 2 Department of Cancer Biology, University of Kansas Medical Center, Kansas City, KS USA

**Keywords:** DNA methylation, Microarray data analysis, Statistical power, Sample size calculation, Bioconductor package, Illumina human methylation BeadChip

## Abstract

**Background:**

When designing an epigenome-wide association study (EWAS) to investigate the relationship between DNA methylation (DNAm) and some exposure(s) or phenotype(s), it is critically important to assess the sample size needed to detect a hypothesized difference with adequate statistical power. However, the complex and nuanced nature of DNAm data makes direct assessment of statistical power challenging. To circumvent these challenges and to address the outstanding need for a user-friendly interface for EWAS power evaluation, we have developed pwrEWAS.

**Results:**

The current implementation of pwrEWAS accommodates power estimation for two-group comparisons of DNAm (e.g. case vs control, exposed vs non-exposed, etc.), where methylation assessment is carried out using the Illumina Human Methylation BeadChip technology. Power is calculated using a semi-parametric simulation-based approach in which DNAm data is randomly generated from beta-distributions using CpG-specific means and variances estimated from one of several different existing DNAm data sets, chosen to cover the most common tissue-types used in EWAS. In addition to specifying the tissue type to be used for DNAm profiling, users are required to specify the sample size, number of differentially methylated CpGs, effect size(s) (Δ_*β*_), target false discovery rate (FDR) and the number of simulated data sets, and have the option of selecting from several different statistical methods to perform differential methylation analyses. pwrEWAS reports the marginal power, marginal type I error rate, marginal FDR, and false discovery cost (FDC). Here, we demonstrate how pwrEWAS can be applied in practice using a hypothetical EWAS. In addition, we report its computational efficiency across a variety of user settings.

**Conclusion:**

Both under- and overpowered studies unnecessarily deplete resources and even risk failure of a study. With pwrEWAS, we provide a user-friendly tool to help researchers circumvent these risks and to assist in the design and planning of EWAS.

**Availability:**

The web interface is written in the R statistical programming language using Shiny (RStudio Inc., 2016) and is available at https://biostats-shinyr.kumc.edu/pwrEWAS/. The R package for pwrEWAS is publicly available at GitHub (https://github.com/stefangraw/pwrEWAS).

**Electronic supplementary material:**

The online version of this article (10.1186/s12859-019-2804-7) contains supplementary material, which is available to authorized users.

## Background

Epigenome-wide association studies (EWAS) aim to examine the relationship between epigenetic marks and exposure(s) or phenotype(s) on a genome-wide level. DNA methylation (DNAm) is the most widely studied epigenetic mechanism and involves the chemical addition of a methyl group to the 5-carbon position of cytosine in the context of cytosine-phosphate-guanine (CpG) dinucleotides. The vast majority of EWAS use microarray-based platforms for assessing DNAm, such as the Illumina Infinium HumanMethylation BeadArrays (Illumina Inc.), as these platforms provide a compromise between coverage, cost, and sample throughput [1, 2]. Illumina’s latest methylation microarrays, the Infinium HumanMethylation450 and Infinium HumanMethylationEPIC, interrogate the methylation levels of over 450,000 and 850,000 CpG dinucleotides, respectively. While these arrays differ in their coverage, both allow for the assessment of methylation at single-nucleotide resolution, quantified using what is referred to as the methylation *β*-value, an approximately continuously-distributed measure that reflects the methylation extent of a specific CpG locus; ranging from 0 (unmethylated) to 1 (methylated). Interest in studying DNAm in the context of human health and disease has been ignited by the now numerous studies that have reported altered patterns of DNAm across various human diseases [3, 4] and in response to environmental exposures [5], along with reversible nature of DNAm, which makes it a promising target for potential treatments and therapies [6]. To detect a hypothesized difference in DNAm with adequate statistical power it is crucial to assess the required sample size. However, the complex nature of DNAm data [7, 8] makes a direct power assessment challenging, as power depends on several factors: planned study sample size, array technology used to profile DNAm, tissue type used in assessing DNAm, proportion of differentially methylated CpGs and the distribution of their differences (Δ_*β*_), and multiplicity.

The importance of formal power assessment and sample size justification in the design of research studies has been recognized and addressed in related omics fields, and motivated the development of power evaluation tools, including: “RNAseqPS” [9], “RNASeqPowerCalculator” [10] and “PROPER” [11] for RNA-Seq data, and “CaTs” [12], “Statistical Power Analysis tool” [13], “GWAPower” [14], and “SurvivalGWAS_Power” [15] for GWAS data. However, surprisingly little attention has been given to this topic in the context of EWAS and while there has been substantial work on the development of statistical methods and publicly available software for the preprocessing, quality control, normalization, and analysis of DNA methylation data [16, 17], methods and tools for power evaluation for EWAS are lagging. Consequently, most EWAS are conducted in the absence of formal power analyses, resulting in studies that are potentially under- or overpowered [18]. To our knowledge, only three studies have formally addressed the issue of power evaluation in the context of EWAS [19–21]. Wang et al. [21] simulated DNAm data for two group comparisons from uniform-normal mixture distributions with parameter settings that capture three general types of distributions often seen in methylation data (methylated, unmethylated, and partially methylated). Power was then assessed and compared for two differential methylation detection methods: proposed method by Wang et al. [21] and t-tests. Rakyan et al. [20] generated DNAm data for two group comparisons from single and mixture beta distributions in three scenarios with four effect sizes each and differences in methylation ranging from 1.25 to 14.4%. Logistic regression was then applied to assess differential methylation and power was evaluated. Finally, Tsai et al. [19] simulated DNAm data for two group comparisons from nine single locus DNAm distributions, again falling into three categories: methylated, hemi-methylated and unmethylated. The expected differences in methylation ranged from 1 to 60%. Differential methylation was then analyzed by t-tests and Wilcoxon rank-sum tests, and the respective power was assessed.

All three approaches utilize a limited number of single locus distributions, which result in a wide range of methylation levels of CpG sites, but may lead to unrealistic data with a predefined fixed number of expected differences in methylation between two groups. This is because individual CpGs have their own unique mean and variance depending on their genomic context and susceptibility to become methylated and vary depending on the tissue type used for methylation assessment [22]. Analogously, expected differences in CpG-specific methylation between two or more groups are expected to come from a continuous distribution instead of having predefined discrete values [23]. In addition to the potential limitations above, none of the previously described methods provided accompanying software for their methodology, limiting their application within the epigenomics-research community. Therefore, there remains an outstanding need for publicly available software that addresses these limitations and enables comprehensive assessments of statistical power in the context of EWAS involving CpG-specific comparisons of DNAm.

Inspired by PROPER [11], a publicly available tool to assist researchers with power assessment in RNA-seq studies, we have developed pwrEWAS for comprehensive power evaluation in the context of case-control EWAS. In pwrEWAS, power is estimated using semi-parametric simulation-based approach. First, DNAm data is randomly generated for each comparator group based on user-supplied information concerning the expected fraction of differentially methylated CpGs between groups and their expected effect size (Δ_*β*_). To simulate realistic methylation data, DNAm data are generated from a beta-distribution using CpG-specific means and variances estimated from one of several different publicly available DNAm data sets, chosen to span the most common tissue-types used in EWAS. This gives the user the flexibility to select the tissue type (e.g., whole blood, peripheral blood mononuclear cells (PBMCs), etc.) that is most appropriate for the study being planned. Next, the generated data undergoes a formal differential methylation analysis, the results of which are used to estimate statistical power. In what follows, we begin by describing the statistical framework underlying pwrEWAS, followed by its demonstration and an assessment of its run time across different user settings. We finish with a discussion of the limitations of pwrEWAS and describe future extensions.

## Methods

As previously mentioned, the Illumina Infinium HumanMethylationEPIC microarray measures the methylation status of > 850,000 CpGs throughout the genome. For a single CpG, DNAm is quantified via the *β*-value, $\beta =\frac{M}{M+U}$, where *M* and *U* are the methylated and unmethylated signal intensities, respectively. As *M* and *U* are typically assumed to be gamma-distributed random variables with equal scale parameter [7], it follows that the *β*-value follows a beta-distribution. As such, the *β*-value ranges from 0 to 1 and represents the methylation extent for a specific CpG. Under ideal conditions, a *β*-value of zero signifies that all alleles in all cells of a sample were unmethylated at that CpG site, while a *β*-value of one indicates methylation throughout all alleles in all cells at that CpG site [24]. A common goal of EWAS is to identify CpG-specific differential methylation based on some phenotype or exposure. Formally, this involves testing the null hypothesis *H*_0_ : Δ_*β*, *j*_ = 0, where ${\Delta}_{\beta, j}={\mu}_j^{(1)}-{\mu}_j^{(2)}$ and represents the difference in mean methylation at the *j*^th^ CpG between two groups (e.g. cases versus controls, exposed versus unexposed, etc.), with *j* = {1,  … , *J*} and *J* representing the number of interrogated CpGs.

**Fig. 1:**
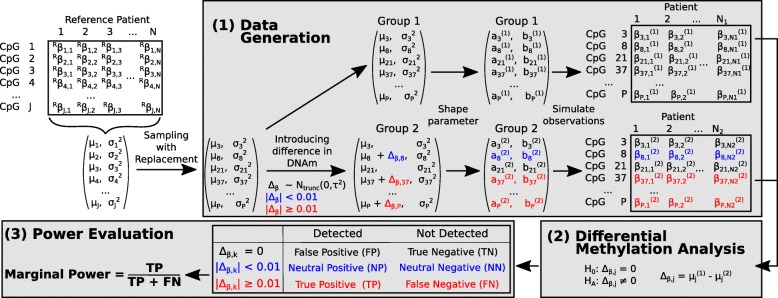
Workflow for pwrEWAS. From an existing tissue-type-specific data set, *J* CpG-specific means and variances are estimated. Next, *P* CpGs are sampled with replacement from the collection of CpGs. For two groups, the mean of one group is changed by *Δ*_*β*_, while the mean of the other group remains unchanged. *Δ*_*β*_ comes from a truncated normal distribution *N*(0, *τ*^2^). These parameters are then used to simulate *β*-values for the two groups. A CpG with an absolute difference in mean methylation greater than a predefined detection limit (default: 0.01) is considered as truly differentially methylated. Next, the simulated data set is used to test for differential, comparing the mean methylation signatures between the two groups. A CpG is defined as “detected” if its corresponding FDR is smaller than a predefined threshold (default: 0.05). Each CpG can fall into one of six categories described in Table 1. The marginal power is calculated as the proportion of True Positives among all truly differentially methylated CpGs

pwrEWAS is written using the R statistical programming language (http://r-project.org) and is comprised of three major steps: (1) data generation, (2) differential methylation analysis, and (3) power evaluation (Fig. 1). Users are required to provide input parameters, including: tissue type to be used for methylation assessment, assumed total sample size (can be specified as a range of possible sample sizes), percentage of the total sample split into two groups (50% corresponds to a balanced study), number of CpGs to be formally tested, expected number of differentially methylated CpGs, and the expected difference in methylation between the comparator groups (Δ_β_) or alternatively, the standard deviation of these differences (*sd*(Δ_*β*_)).

To assist users with their experimental design, pwrEWAS provides estimates of statistical power as a function of the assumed sample and effect size(s). Further, it provides estimates of the marginal type I error rate, marginal FDR, false discovery cost (FDC), the distribution of simulated Δ_*β*_ ’s, and probabilities of identifying at least one true positive. The probability of identifying at least one true positive is beneficial in studies where either the effect or sample size is very small (e.g. pilot or explanatory studies).

### Data generation

Our approach to estimating statistical power begins by leveraging publicly available DNA methylation data sets in order to simulate realistic methylation data. Data sets used for the purpose of simulation were selected to represent the most commonly used tissue types used in EWAS. To identify these tissue types, the Gene Expression Omnibus (GEO) data repository was manually scanned and tissue types were rank-ordered based on the number of GEO deposited data sets including Illumina Infinium Human Methylation BeadChip data for that tissue type. For each of the most common tissue types identified, a single representative data set was selected (Table 1). Representative datasets were selected based on a combination of the study’s sample size (preference toward larger data sets), study design, and the inclusion of DNA methylation profiles for healthy, non-diseased subjects.

**Table 1:** Curated tissue-type specific DNAm data sets used by pwrEWAS

Tissue Type	Accession Number	Subjects within GSE-ID limited to	Reference
Saliva	GSE92767		[38]
Lymphoma	GSE42372	disease state: non-HIV lymphoma	[39]
Placenta	GSE62733	health state: Normal	[40]
Liver	GSE61258	diseasestatus: Control	[41]
Colon	GSE77718	disease state: Normal	[42]
Blood (Adults)	GSE42861	subject: Normal	[43, 44]
Blood (Children)	GSE83334	age: 5 years	[45]
Blood (Newborns)	GSE82273		[46]
Cord-blood (whole blood)	GSE69176		
Cord-blood (PBMC)	GSE110128	cord blood	[47]
Adult (PBMC)	GSE67170	disease state: control	[48]

Representative data sets for the most commonly used tissue types for EWAS with inclusion criteria for subjects

For each selected tissue type, CpG-specific means and variances were estimated (${\widehat{\mu}}_j=\frac{1}{N}{\sum}_{i=1}^N{\beta}_{i,j}$ and ${\hat{\sigma}}_j^2=\frac{1}{N-1}{\sum}_{i=1}^N{\left({\beta}_{i,j}-{\hat{\mu}}_j\right)}^2$), where *β*_*i*, *j*_ represents the methylation *β*-value for CpG *j* = {1,  … , *J*} in subject *i* = {1,  … , *N*}. CpG-specific parameter estimates are then used as the basis for simulating realistic methylation data using a semi-parametric simulation strategy. First, *P* pairs of CpG-specific means and variances $\left({\widehat{\mu}}_j,{\widehat{\sigma}}_j^2\right)$ are sampled with replacement from one of the tissue-type specific reference data sets (Table 1). By default, *P* is set to 100,000 CpG sites, as previous studies have suggested filtering out low-variable CpGs to offset the burden of multiplicity [25], however in principle, *P* can be set according to the user’s preference (e.g., *P* = 866,836 for EWAS conducted using the EPIC array). Thus, pwrEWAS allows up- or down-scaling to any number of CpGs that the investigator plans to measure and conducted differential methylation analyses on. This is an important feature since the EPIC array is the successor to the now discontinued Infinium HumanMethylation450 array, which represents the technology used for methylation assessment of the tissue-specific reference data sets used as the basis of our simulation strategy. Of the *P* sampled CpGs, a difference in mean DNAm (Δ_*β*_) is imposed on *K* CpGs, where *K* ≤ *P*. The number of differentially methylated CpGs, *K*, is selected by the user and ideally motivated by a pilot study, previous literature, or expert knowledge about the effect of the phenotype(s) or exposure(s) of interest on DNA methylation. The mean methylation of *K* CpGs is shifted in one of the comparator groups by Δ_*β*_ = {Δ_*β*, 1_, …Δ_*β*, *k*_, …Δ_*β*, *K*_}, while the mean methylation in the other comparator group remains unchanged. Due to the nature of *β*-values and the parameter restrictions of the beta distribution (0 ≤ *μ*_*k*_ ≤ 1 and $0<{\sigma}_k^2<0.25$), Δ_*β*, *k*_ is bounded by $\frac{1}{2}-{\mu}_k\pm \sqrt{\frac{1}{4}-{\sigma}_k^2}$, where *μ*_*k*_ and ${\sigma}_k^2$ are CpG-specific means and variances, respectively (see Additional file 1 for additional details). Due to its boundedness, Δ_*β*, *k*_ is drawn from a truncated normal distribution (Δ_*β*, *k*_~*N*_*k*_(0, *τ*^2^)). The normal distribution was chosen based on observed differences in DNAm of differentially methylated CpGs in previously published EWAS (see Additional file 2: Figure S1). The standard deviation of the simulated differences *τ* can be provided by the user or be automatically be determined based on the user-specified target Δ_*β*_ and the expected number of differentially methylated CpGs, such that Δ_*β*_ matches the target maximal difference in mean methylation. To achieve this, an internal function simulates *P* Δ_*β*, *k*_ ’s (this matches the number of subsequently simulated CpGs) 100 times, while stepwise adjusting *τ*. The goal is to identify a standard deviation *τ* for the truncated normal distribution to matches the targeted maximal difference in DNAm. Therefore, *τ* is adjusted stepwise until the 99.99th percentile of the absolute value of simulated Δ_*β*, *k*_ ’s falls within a range around the targeted maximal difference in DNAm. The range is equal to the detection limit (±0.005 based on default detection limit: 0.01). (Additional file 2: Figure S2) shows the distribution of simulated Δ_*β*, *k*_ ’s for different effect sizes and its respective range that the 99.99th percentile of the simulated Δ_*β*, *k*_ ’s needs to fall in for *τ* to be accepted.

Since Δ_*β*_ is simulated from a truncated normal distribution, a certain proportion of Δ_*β*_ are within the detection limit range around zero and thus, do not exhibit a biologically meaningful difference in mean methylation. To ensure that *K* includes the number of meaningfully differential methylated CpGs (truly differentially methylated CpGs), *K* is calculated to reflect the user-supplied target number of differentially methylated CpGs ($K=\frac{1}{Percentage\ of\ truly\  DM\  CpGs}\ast Target\ number\ of\  DM\  CpGs$). This results in *K* CpGs with changed means (Δ_*β*, *k*_ ≠ 0) and *P* − *K* CpGs with unchanged means (Δ_*β*, *k*_ = 0) between the two comparator groups. Variances across all *P* CpGs remain unchanged in both comparator groups, that is, comparator groups are assumed to have the same CpG-specific variances. Next, the means and variances of both comparator groups are used to calculate CpG-specific shape parameters for the beta-distribution: ${a}_j={\mu}_j^2\left(\frac{1-{\mu}_j}{\sigma_j^2}-\frac{1}{\mu_j}\right)$ and ${b}_j={a}_j\left(\frac{1}{\mu_j}-1\right)$ (see Additional file 1). The two comparator group specific matrices (*P* × *2*) containing the CpG-specific shape parameters are then used to generate *N*_1_ and *N*_2_ beta-distributed observations for each CpG, for both comparator groups respectively, resulting in two matrices (*P* x *N*_1_ and *P* x *N*_2_) of *β*-values, which are subsequently used for the differential methylation analysis.

Simulated CpGs fall into one of three categories: (1) not differentially methylated (Δ_*β*, *k*_ = 0), (2) differentially methylated with negligible difference (|Δ_*β*, *k*_| < 0.01), and (3) truly differentially methylated (|Δ_*β*, *k*_| ≥ 0.01). The threshold of 0.01 was chosen according to the detection limit of DNAm arrays [8], but can be modified by the user.

### Differential methylation detection

Following data generation, differential methylation analyses are carried out using one of several established parametric and nonparametric approaches, including: limma [26], CpGassoc [27], t-test, or a Wilcoxon rank-sum test. In the first three of the above methods, simulated *β*-values are first transformed to methylation *M*-values using the logit-transformation ($M={\log}_2\left(\frac{\beta }{1-\beta}\right)$) due to their assumption of normality [24, 28]. Each method reports CpG-specific *p*-values, which are multiplicity adjusted using the Benjamini and Hochberg method [29] to control the False Discovery Rate (FDR).

### Power assessment

Tested CpGs fall into one of six categories: (1) TP (True Positive): detected CpGs with meaningful difference in mean DNAm, (2) NP (Neutral Positive): detected CpGs with negligible difference in mean DNAm, (3) FP (False Positive): detected CpG with no difference in mean DNAm, (4) TN (True Negative): undetected CpGs with no difference in mean DNAm, (5) NN (Neutral Negative): undetected CpGs with negligible difference in mean DNAm, and (6) FN (False Negative): undetected CpGs with meaningful difference in mean DNAm (Table 2).

**Table 2:** Differential methylation detection and terminology

	Differentially Methylated	Truly Differentially Methylated	Detected	Not Detected
Δ_*k*_ = 0	No	No	False Positive (FP)	True Negative (TN)
|Δ_*k*_| < 0.01	Yes	No	Neutral Positive (NP)	Neutral Negative (NN)
|Δ_*k*_| ≥ 0.01	Yes	Yes	True Positive (TP)	False Negative (FN)

Each CpG can fall into one of six following categories: False Positive (FP; detected CpG with no simulated difference in mean methylation); Neutral Positive (NP; detected CpG with negotiable simulated difference in mean methylation); True Positive (TP; detected CpG with meaningful simulated difference in mean methylation); True Negative (TN; not detected CpG with no simulated difference in mean methylation); Neutral Negative (NN; not detected CpG with negotiable simulated difference in mean methylation); False Negative (FN; not detected CpG with meaningful simulated difference in mean methylation)

Since it can be argued that CpGs with a negligible Δ_*β*, *k*_ are not biologically meaningful, we calculate the empirical marginal power, defined by Wu et al. [11] as the proportion of truly differentially methylated CpGs detected at the specified FDR threshold, $\frac{TP}{TP+ FN}$ (Table 2). Further, even though failing to discover differentially methylated CpGs represents a type II error, failing to detect CpGs with a negligible Δ_*β*, *k*_ can be disregarded (NN) due to their likely unimportance. Additionally, as identifying CpGs with a negligible Δ_*β*, *k*_ (NP) is not as crucial as identifying CpGs with a biologically meaningful Δ_*β*, *k*_ (TP), we also report the false discovery cost ($FDC=\frac{FP}{TP}$) [11].

For each of the assumed sample and effect sizes we report the following metrics, averaged across simulations to obtain reliable estimates:

- **Empirical classical power:** The ratio of correctly detected CpGs and all differentially methylated CpGs

$$ classicalPower=\frac{NP+ TP}{NP+ NN+ TP+ FN} $$

- **Empirical marginal power:** The ratio of correctly detected CpGs with biologically meaningful differences and all differentially methylated CpGs with biologically meaningful differences (excluding Neutral Positives and Neutral Negative with negligible differences):

$$ marPower=\frac{TP}{TP+ FN} $$

- **Empirical marginal Type I Error:** The ratio of wrongly detected CpGs and all CpGs with no difference

$$ marTypeI=\frac{FP}{FP+ TN} $$

- **Empirical False Discovery Rate (FDR):** The ratio of wrongly detected CpGs and all detected CpGs

$$ FDR=\frac{FP}{FP+ NP+ TP} $$

- **Empirical False Discovery cost (FDC):** The ratio of wrongly detected CpGs and correctly detected CpGs:

$$ FDC=\frac{FP}{TP} $$

### Visualization

**Fig. 2:**
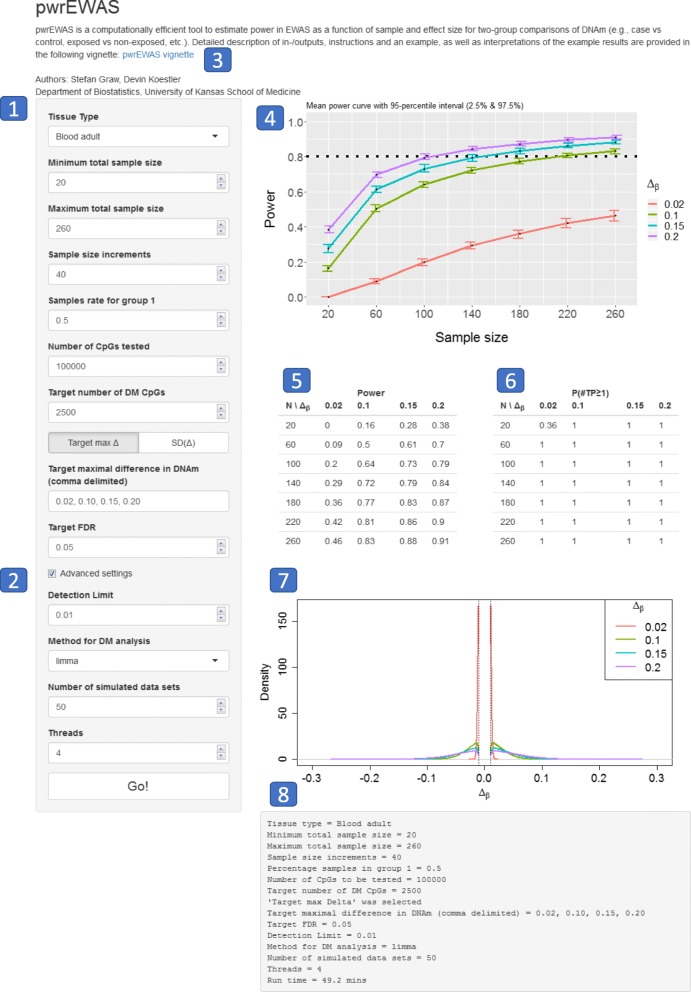
pwrEWAS Shiny User-Interface. (1) User-specific inputs; (2) Advanced input settings to optimize run time; (3) Link to vignette for detailed description of inputs and outputs, instructions and an example including interpretations of the example results; (4) Power curve as a function of sample size by effect size (Δ_*β*_); (5) Estimated power average over simulation by sample size and effect size (Δ_*β*_); (6) Probability of detection at least one true positive; (7) Distribution of simulated differences in DNAm (Δ_*β*_) for different target Δ_*β*_ ’s; (8) Log of input parameter and run time

The pwrEWAS package contains two functions that can be used to visualize the results (“pwrEWAS_powerPlot” and “pwrEWAS_deltaDensity”). “pwrEWAS_powerPlot” displays the estimated power as a function of sample size with error bars (2.5th and 97.5th percentile calculated across simulations). Power across different target Δ_β_^′^ s as a function of sample size is differentiated by different colors (Fig. 2, Box 4). “pwrEWAS_deltaDensity” illustrates the distribution of simulated Δ_β, k_^′^ s for different target Δ_β_^′^ s as density plots (Fig. 2**,** Box 7). Densities for different target Δ_β_^′^ s are color-coded as well and match the colors of the power curve (“pwrEWAS_powerPlot”).

## Results

Consider a hypothetical study that aims to understand the relationship between electronic cigarettes (e-cigarette) and DNAm derived from adult blood. The use of e-cigarettes has increased dramatically over the last decade, especially among young adults [30]. There exists a common perception in the population, including pregnant women and women in child-bearing age, that e-cigarettes are less harmful than smoking tobacco cigarettes [31]. Although, studies have reported the presence of toxic components in e-cigarette aerosol [30], there presently exists no study investigating the relationship between e-cigarette and DNAm derived from adult human blood. As the effect of e-cigarette usage on DNAm is presently unknown, but is of interest in this hypothetical study, we will use the previously reported effects of tobacco smoke on blood-derived DNAm as an upper limit for the effect of e-cigarette usage on DNAm. Previous studies analyzing the effect of smoking tobacco cigarettes on blood-derived patterns of DNA methylation have reported CpG-specific differences up to 24% between smokers and non-smokers, with a wide range of CpGs (724–18,760) declared as significantly differentially methylated (FDR ≤0.05) [32–34]. Hence, we want to investigate the number of subjects required to detect DNAm differences in 2500 CpGs (selected to be within the range of the number of significantly differently methylated CpGs reported between smokers and non-smokers in previous reports) with 80% power for three reasonable effect sizes (Δ_*β*_ = {0.10, 0.15, 0.20} and one deliberately small effect size Δ_*β*_ = 0.02, representing differences in DNAm up to ~2 % , ~10 % , ~15% and ~20%). To cover a wide range of total sample sizes, we analyzed total sample sizes ranging from 20 to 260 individuals with increments of 40 and equal allocation between e-cigarette users and non-users, while keeping the remaining default parameters of pwrEWAS intact:

- Tissue type: Blood adult
- Minimum total sample size: 20
- Minimum total sample size: 260
- Sample size increments: 40
- Samples rate for group 1: 0.50
- Number of CpGs tested: 100000
- Target number of DM CpGs: 2500
- Select ‘Target max Δ ’
- Target maximal difference in DNAm: 0.02, 0.10, 0.15, 0.20
- Target FDR: 0.05
- Detection Limit: 0.01
- Method for DM analysis: limma
- Number of simulated data sets: 50
- Threads: 4

The results of this power analysis can be found in Fig. 2. To detect differences up to 10, 15 and 20% in CpG-specific methylation across 2500 CpGs between e-cigarette users and non-users with at least 80% power, we would need about 220, 180 and 140 total subjects, respectively. As expected, 80% power was not achieved for a difference in DNAm ≤2% for the selected total sample size range. However, it can be observed for this target differences of 2%, that the probability of detecting at least one CpG out of the 2500 differentially methylated CpGs is about 36% for 20 total patients and virtually 100% for 60 and more total patients. Because there exists no literature on the magnitude of expected differences in DNAm, a pilot study would be helpful in this hypothetical situation to narrow the range of expected differences to more precisely identify the required sample size to achieve 80% power.

To evaluate this broad range of sample and effect sizes of this theoretical experiment, pwrEWAS required ~ 49 min in total. In general, the computational complexity of pwrEWAS depends on four major components: (1) assumed number and magnitude of sample size(s), (2) number of target Δ_*β*_ ’s (effect sizes), (3) number of CpGs tested, and (4) number of simulated data sets. To enhance the computational efficiency, pwrEWAS allows users to process simulations in parallel. While (1) and (2) are usually dictated by the study to be conducted, (3) and (4) can be modified to either increase the precision of power estimates (increased run time) or reduce the computational burden (decreased precision of estimates). The run time of pwrEWAS for different combinations of sample sizes and effect sizes are provided in Table 3.

**Table 3:** Run time of pwrEWAS for different combinations of sample sizes and effect sizes

Total sample sizes	Effect sizes (Δ_*β*_)		
	0.1	0.1, 0.2	0.1, 0.3, 0.5
10	2 min 21 s	3 min 11 s	3 min 50s
100	6 min 22 s	7 min 39 s	8 min 33 s
500	24 min 43 s	27 min 36 s	29 min 22 s
10–100 (increments of 10)	9 min 40s	16 min 34 s	23 min 44 s
300–500 (increments of 100)	27 min 58 s	30 min 01 s	52 min 00s

In all scenarios presented the number of tested CpGs was assumed to be 100,000, number of simulated data sets was 50, and the method to perform the differential methylation analysis as limma. A total of 6 clusters/threads were used

**Fig. 3:**
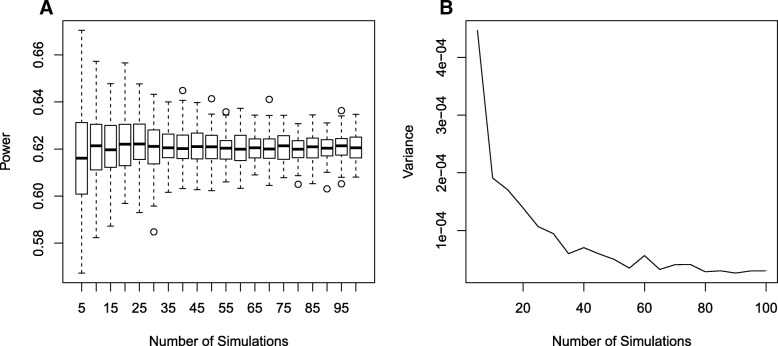
Empirical assessment of the number of simulations. To assess the number of simulated data sets (number of simulations) required to obtain consistent results for power, pwrEWAS was run for a variety of number of simulations (5–100 simulations), each 100 times and each with the same remaining input parameters. **a** shows the distribution of power estimates for 100 runs within each of the assumed number of simulations. **b** visualizes the variance of power estimates for each of the assumed number of simulations. Given the relative stability of variance estimates beyond 50 simulations, 50 was selected as the default value for the number of simulations in pwrEWAS

As the number of simulated data sets is one of the major components (e.g., item (4), above) affecting the run time of pwrEWAS, it is important to identify a default value that offers a reasonable tradeoff between run time and precision of power estimates. To this end, the variance of power estimates was assessed for a range of simulated data sets (5–100), each repeated 100 times, while keeping the remaining parameters unchanged (Fig. 3a). We ultimately determined the default value for the number of simulated data sets to be 50, as it appears that simulating additional data sets reduces the variance of power estimates only marginally (Fig. 3b).

The pwrEWAS package is accompanied by a vignette, which provides a more detailed description of input and output, instructions for the usage, an example, and interpretations of the example results. In addition, a user-friendly R-Shiny point-and-click interface has been developed (Fig. 2) for researchers that are unfamiliar or less comfortable with the R environment.

## Discussion

In our hypothetical study on the effect of e-cigarette usage on patterns of blood-derived DNAm, we found that 140–220 total subjects would be needed, depending on the expected effect size. However, these results should be treated with a certain level of caution and considered to be more of a guideline than an exact prescription. Due to computational, memory and storage burden, and simplicity considerations, pwrEWAS involves the random generation methylation *β*-values independently across CpGs, which might not hold in real data given previous reports of local correlation in DNAm of nearby CpG sites [35]. Additionally, pwrEWAS assumes CpG-specific homoscedasticity between both comparator groups, that is CpG-specific variances are assumed to be identical between both groups. However, CpG-specific variances have been reported to change depending on exposure(s) and phenotype(s) [36, 37]. Violations of CpG-specific homoscedasticity can result in inflated estimates of statistical power and produce overly optimistic sample sizes, however identifying the magnitude of changes in variances depending on exposure(s) and phenotype(s) in advance of the study can be very challenging. Further, the expected difference in DNAm between both groups (Δ_*β*_) is assumed to come from a truncated normal. This assumption seems to hold, at least approximately, based on observed distributions of differences in DNAm across a variety of studies. Additional limitations of pwrEWAS include: two group comparison, selection of methods for differential methylation analysis, and selection of tissue types specific reference data.

Despite the above limitations, pwrEWAS is to our knowledge, the first publicly available tool to formally address the issue of power evaluation in the context of EWAS. Further opportunities for the extension of pwrEWAS include the implementation of additional methods for differential methylation analysis (e.g., linear regression for continuous phenotype(s)/exposure(s), Cox-proportional hazards models or relevant models for handling time-to-event outcomes, etc.), allowing multiple group comparisons, providing the opportunity for researcher to upload different reference data (tissue type(s) specific to their study), and addressing the potential change of CpG dispersion due to phenotype(s) and/or exposure(s).

## Conclusion

When designing an EWAS, consideration of statistical power should play a central role in selecting the appropriate sample size to address the question(s) of interest. Under- and overpowered studies waste resources and even risk failure of the study. With pwrEWAS we present a user-friendly power evaluation tool with the goal of helping researchers in the design and planning of their EWAS.

### Availability and requirements

**Project name:** pwrEWAS.

**Project homepage:** https://github.com/stefangraw/pwrEWAS

**Operating systems:** Platform independent.

**Programming language:** R.

**License:** Artistic-2.0.

**Any restrictions to use by non-academics:** none.

## Additional files

Additional file 1: Derivation for upper and lower bound of Δ, CpG-specific differences in mean methylation between two compared groups. (DOCX 28 kb)

Additional file 2: Supplementary Figure 1 and Supplementary Figure 2. (DOCX 639 kb)

## Abbreviations

DNAm: DNA methylation
EWAS: Epigenome-Wide Association Study
FDC: False Discovery Cost
FDR: False Discovery Rate
