# Supplementary material for: pwrEWAS: a user-friendly tool for comprehensive power estimation for epigenome wide association studies (EWAS)
Source: BMC Bioinformatics. 2019 Apr 29;20:218. doi: 10.1186/s12859-019-2804-7 (PMC6489300; doi:10.1186/s12859-019-2804-7)
Supplement: Supplementary file 1 — Derivation for upper and lower bound of Δ, CpG-specific differences in mean methylation between two compared groups. (DOCX 28 kb) [file 12859_2019_2804_MOESM1_ESM.docx]

**Additional file 1**

**pwrEWAS: A user-friendly tool for comprehensive power estimation in epigenome wide association studies (EWAS)**

Stefan Graw, Rosalyn Henn, Jeffrey A. Thompson, and Devin C. Koestler

# Calculations

PDF of the beta distribution, $Beta\left( \alpha,\beta\right)$, with shape parameter $\alpha>0$ and $\beta>0$:

$$\frac{x^{\alpha-1}\left( 1-x \right)^{\beta-1}}{B\left( \alpha,\beta\right)}$$

Where $B\left( \alpha,\beta\right)=\frac{\Gamma(\alpha)\Gamma(\beta)}{\Gamma(\alpha+\beta)}$

Mean ($\mu$) and variance ($\sigma^{2}$):

|  | $\mu=\frac{\alpha}{\alpha+\beta}$ $\sigma^{2}=\frac{\alpha\beta}{\left( \alpha+\beta\right)^{2}(\alpha+\beta+1)}$ | (1.1)  (1.2) |
| --- | --- | --- |

Shape parameter as function of mean ($\mu$) and variance ($\sigma^{2}$):

$\beta$ as a function of $\alpha$ and $\mu$ from (1.1):

|  | $\mu=\frac{\alpha}{\alpha+\beta}$ $\beta\mu=\alpha-\alpha\mu$ $\beta=\alpha\left( \frac{1}{\mu}-1 \right)$ | (1.3) |
| --- | --- | --- |

$\alpha$ as a function of $\mu$ and $\sigma^{2}$ (using (1.3) in (1.2)):

|  | $\sigma^{2}=\frac{\alpha\beta}{\left( \alpha+\beta\right)^{2}\left( \alpha+\beta+1 \right)}$ $\sigma^{2}=\frac{\alpha^{2}\left( \frac{1}{\mu}-1 \right)}{\left( \alpha+\alpha\left( \frac{1}{\mu}-1 \right) \right)^{2}\left( \alpha+\alpha\left( \frac{1}{\mu}-1 \right)+1 \right)}$  $\sigma^{2}=\frac{\left( \frac{1}{\mu}-1 \right)}{\left( 1+\left( \frac{1}{\mu}-1 \right) \right)^{2}\left( \alpha+\alpha\left( \frac{1}{\mu}-1 \right)+1 \right)}$ $\alpha+\alpha\left( \frac{1}{\mu}-1 \right)+1=\frac{\left( \frac{1}{\mu}-1 \right)}{\sigma^{2}\left( \frac{1}{\mu} \right)^{2}}$ $\alpha\left( \frac{1}{\mu} \right)=\frac{\left( \frac{1}{\mu}-1 \right)\mu^{2}}{\sigma^{2}}-1$ $\alpha=\mu^{2}\left( \frac{1-\mu}{\sigma^{2}}-\frac{1}{\mu} \right)$ | (1.4) |
| --- | --- | --- |

$\beta$ as a function of $\mu$ and $\sigma^{2}$ (using (1.4) in (1.3)):

|  | $\beta=\mu^{2}\left( \frac{1-\mu}{\sigma^{2}}-\frac{1}{\mu} \right)\left( \frac{1}{\mu}-1 \right)$ | (1.5) |
| --- | --- | --- |

Relationship between $\mu$ and $\sigma^{2}$:

|  | $\alpha>0$ $\mu^{2}\left( \frac{1-\mu}{\sigma^{2}}-\frac{1}{\mu} \right)>0$ $\left( \frac{1-\mu}{\sigma^{2}}-\frac{1}{\mu} \right)>0$ $\frac{1-\mu}{\sigma^{2}}>\frac{1}{\mu}$ $\mu-\mu^{2}>\sigma^{2}$ | (2.1) |
| --- | --- | --- |

Consider a modified mean $\mu^{*}=\mu+\Delta$, where the original mean $\mu$ was changed by $\Delta$, while $\sigma^{2}$ remains unchanged. This modified mean $\mu^{*}$ must satisfy relationship (2.1), too:

|  | $\mu^{*}=\mu+\Delta$  $\mu^{*}-{\mu^{*}}^{2}>\sigma^{2}$ $\mu+\Delta-\left( \mu+\Delta\right)^{2}>\sigma^{2}$ $-\mu-\Delta+\left( \mu+\Delta\right)^{2}<-\sigma^{2}$ $-\Delta+\mu^{2}+2\mu\Delta+\Delta^{2}<\mu-\sigma^{2}$ $\Delta^{2}+2\Delta\left( \mu-\frac{1}{2} \right)<\mu-\mu^{2}-\sigma^{2}$ $\Delta^{2}+2\Delta\left( \mu-\frac{1}{2} \right)+\left( \mu-\frac{1}{2} \right)^{2}<\mu-\mu^{2}-\sigma^{2}+\left( \mu-\frac{1}{2} \right)^{2}$ $\left( \Delta+\left( \mu-\frac{1}{2} \right) \right)^{2}<\mu-\mu^{2}-\sigma^{2}+\mu^{2}-\mu+\frac{1}{4}$ $\Delta+\left( \mu-\frac{1}{2} \right)<\pm\sqrt{\frac{1}{4}-\sigma^{2}}$ $\Delta<\frac{1}{2}-\mu\pm\sqrt{\frac{1}{4}-\sigma^{2}}$ | (2.2) |
| --- | --- | --- |

Hence, the imposed difference $\Delta$ is bounded by:

|  | $\frac{1}{2}-\mu-\sqrt{\frac{1}{4}-\sigma^{2}}<\Delta<\frac{1}{2}-\mu+\sqrt{\frac{1}{4}-\sigma^{2}}$ | (2.3) |
| --- | --- | --- |
