# Supplementary material for: pwrEWAS: a user-friendly tool for comprehensive power estimation for epigenome wide association studies (EWAS)
Source: BMC Bioinformatics. 2019 Apr 29;20:218. doi: 10.1186/s12859-019-2804-7 (PMC6489300; doi:10.1186/s12859-019-2804-7)
Supplement: Supplementary file 2 — Supplementary Figure 1 and Supplementary Figure 2. (DOCX 639 kb) [file 12859_2019_2804_MOESM2_ESM.docx]

**Additional file 2**

**pwrEWAS: A user-friendly tool for comprehensive power estimation in epigenome wide association studies (EWAS)**

Stefan Graw, Rosalyn Henn, Jeffrey A. Thompson, and Devin C. Koestler

# Figures





**Supplementary Figure 1:** **Empirical distributions of differences in DNAm for CpGs identified in different studies.** Density plots showing the distribution of differences in DNAm for CpGs identified in the following three studies: PMC6084815 [1]{Hannon, 2018 #53}, PMC4256841 [2], and PMC4864062 [3]. The first study (PMC6084815) reported the differences in DNAm ($\Delta_{\beta}$) of 20,001 differentially methylated CpGs (p-value < $1x{10}^{-8}$) comparing the additive genetic effects between monozygotic and dizygotic twins. The second study (PMC4256841) identified 470 differentially CpGs specific to the sex in human pancreatic islets. Difference in DNAm greater than 0.05 with a FDR less than 5% ($q<0.05$) were provided. In the third study (PMC4864062) two normal tissues were discriminated based on their methylation profile: colon mucosa (NC22A) and neurons (N229). Differences in DNAm greater than 0.33 were reported for 73,774 CpGs. It becomes apparent that simulating differences in DNAm from a truncated normal distribution, where values around zero (detection limit) are omitted, imitates observed values reasonable well. The truncation of the normal distribution is required due to the support of $\Delta_{\beta}$ ($-1\leq\Delta_{\beta}\leq1$). Even though the shown distributions exhibit some imbalance between positive and negative values, it is not necessary to preserve this potential imbalance as it will not affect the estimated power.


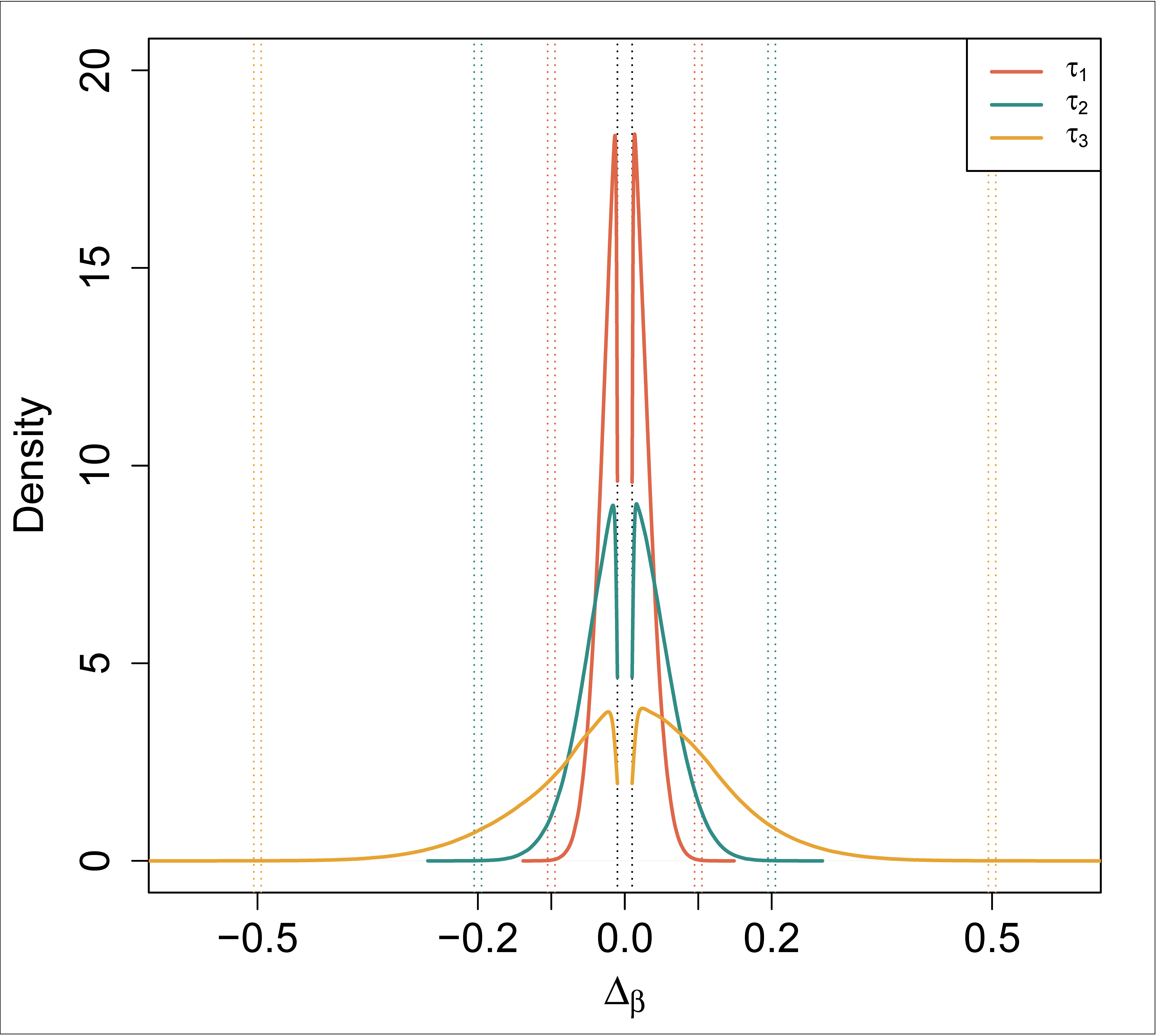


**Supplementary Figure 2: Distributions of simulated differences in DNAm (**$\boldsymbol{\Delta}_{\boldsymbol{\beta}}$**) for different target effect sizes.** $\Delta_{\beta}$ is simulated using a truncated normal distribution $\Delta_{\beta,k}\sim N_{k}(0,\tau)$. To match the targeted maximal difference in DNAm, $\tau$ is stepwise adjusted until the 99.99^th^ percentile of the absolute value of simulated $\Delta_{\beta,k}$’s falls within a range (vertical colored dotted lines) around the targeted maximal difference in DNAm. The range is equal the detection limit (vertical black dotted lines). Based on a default detection limit of 0.01, the 99.99^th^ percentile needs to fall within a target effect size $\pm0.005$ for a $\tau$ to be accepted. The figure above shows densities of simulated $\Delta_{\beta,k}$’s for three effect sizes (0.1, 0.2, 0.5), and range for each effect size that the 99.99^th^ percentile is required to fall in for $\tau$ to be accepted.

# References

1. Hannon E, Knox O, Sugden K, Burrage J, Wong CCY, Belsky DW, Corcoran DL, Arseneault L, Moffitt TE, Caspi A *et al*: **Characterizing genetic and environmental influences on variable DNA methylation using monozygotic and dizygotic twins**. *Plos Genet* 2018, **14**(8).

2. Hall E, Volkov P, Dayeh T, Esguerra JLS, Salo S, Eliasson L, Ronn T, Bacos K, Ling C: **Sex differences in the genome-wide DNA methylation pattern and impact on gene expression, microRNA levels and insulin secretion in human pancreatic islets**. *Genome Biol* 2014, **15**(12).

3. Moran S, Arribas C, Esteller M: **Validation of a DNA methylation microarray for 850,000 CpG sites of the human genome enriched in enhancer sequences**. *Epigenomics* 2016, **8**(3):389-399.
